# Cellular Proliferation, Dermal Repair, and Microbiological Effectiveness of Ultrasound-Assisted Wound Debridement (UAW) Versus Standard Wound Treatment in Complicated Diabetic Foot Ulcers (DFU): An Open-Label Randomized Controlled Trial

**DOI:** 10.3390/jcm9124032

**Published:** 2020-12-13

**Authors:** José Luis Lázaro-Martínez, Francisco Javier Álvaro-Afonso, David Sevillano-Fernández, Yolanda García-Álvarez, Irene Sanz-Corbalan, Esther García-Morales

**Affiliations:** 1 Diabetic Foot Unit, Clínica Universitaria de Podología, Facultad de Enfermería, Fisioterapia y Podología, Universidad Complutense de Madrid, 28040 Madrid, Spain; diabetes@ucm.es (J.L.L.-M.); ygarci01@ucm.es (Y.G.-Á.); irsanz01@ucm.es (I.S.-C.); eagarcia@ucm.es (E.G.-M.); 2 Instituto de Investigación Sanitaria del Hospital Clínico San Carlos (IdISSC), 28040 Madrid, Spain; 3 Microbiology Section, Facultad de Medicina, Universidad Complutense de Madrid, 28040 Madrid, Spain; dsevill@med.ucm.es

**Keywords:** ultrasound assisted wound debridement, surgical debridement, cellular proliferation, microbiology, diabetic foot ulcers

## Abstract

We aimed to evaluate the effects of ultrasound-assisted wound (UAW) debridement on cellular proliferation and dermal repair in complicated diabetic foot ulcers as compared to diabetic foot ulcers receiving surgical/sharp wound debridement. A randomized controlled trial was performed involving 51 outpatients with complicated diabetic foot ulcers that either received surgical debridement (*n* = 24) or UAW debridement (*n* = 27) every week during a six-week treatment period. Compared to patients receiving surgical debridement, patients treated with UAW debridement exhibited significantly improved cellular proliferation, as determined by CD31 staining, Masson’s trichrome staining, and actin staining. Bacterial loads were significantly reduced in the UAW debridement group compared to the surgical group (UAW group 4.27 ± 0.37 day 0 to 2.11 ± 0.8 versus surgical group 4.66 ± 1.21 day 0 to 4.39 ± 1.24 day 42; *p* = 0.01). Time to healing was also significantly lower (*p* = 0.04) in the UAW group (9.7 ± 3.8 weeks) compared to the surgical group (14.8 ± 12.3 weeks), but both groups had similar rates of patients that were healed after six months of follow-up (23 patients (85.1%) in the UAW group vs. 20 patients (83.3%) in the surgical group; *p* = 0.856). We propose that UAW debridement could be an effective alternative when surgical debridement is not available or is contraindicated for use on patients with complicated diabetic foot ulcers.

## 1. Introduction

Standard of care in patients with diabetic foot ulcers includes pressure off-loading, treatment of infection, restoration of tissue perfusion, metabolic control of diabetes, treatment of co-morbidities and local ulcer care [1].

Wound debridement is a fundamental part of wound bed preparation (WBP) during diabetic foot ulcer (DFU) treatment. Regular wound debridement helps to eliminate biofilms from the wound bed, as well as remove necrotic tissue that may favor biofilm re-growth [2,3].

While surgical debridement is considered the gold standard in DFU treatment and should be utilized over other techniques, it is not always available, practical or suitable or for each patient [4,5]. When considering co-morbidities, vascular status, level of infection, ulcer location, and patient preference, practitioners may find that alternative debridement methods are more appropriate as the primary treatment or in tandem with other treatments over time. Likewise, surgical debridement has certain limitations: it is not ideal for patients with poor vascular status; it requires specific surgical skills; for the procedure is required an operating room; and surgical debridement has the potential for large damage to wound beds with exposure bone, joint tissue or ligament [6]. 

According to a recent panel’s clinical experiences held in Berlin 2018, when surgical debridement is not available for use or contraindicated on DFUs, an effective alternative is to utilize mechanical debridement strategies such as ultrasonic-assisted wound (UAW) debridement or low-frequency contact ultrasonic debridement (LFCUD) [4]. UAW has proven to be effective in cleaning wounds of non-viable tissue and slough and removing biofilms without damaging healthy, viable tissue by using the micro-streaming and cavitation effects of ultrasound [7]. In this regard, several studies have claimed that ultrasound debridement leads to greater viability of granulation tissue, increasing closure rates in chronic wounds [8,9,10].

A recent single-center, non-comparative study evaluating the microbiological and clinical effects of UAW debridement on patients with neuroischaemic DFU over a six-week treatment period demonstrated that UAW debridement reduces bioburden and biofilm reformation [6]. The findings from this study also indicated that sequential UAW debridement resulted in ongoing reduction of bacterial load, which was correlated with an improvement in the condition of the wound bed, and progressive reduction in wound size over the follow-up period [4,5,6]. The limitations of this previous study were that it did not involve a control group that received the gold standard surgical debridement, and it did not evaluate histological or biochemical features of the tissues. 

In order to build upon these previous findings, the main aim of the current study was to elucidate the effects of UAW debridement on cellular proliferation and dermal repair in complicated diabetic foot ulcers (DFU) as compared to DFUs treated with surgical/sharp wound debridement over a six-week treatment period. The secondary aim of our study was to evaluate the reduction of the bacterial burden from UAW debridement compared to DFUs receiving surgical wound debridement. Finally, the third aim was to evaluate and compare the effects of six-week treatment with UAW debridement or surgical wound debridement on wound condition, wound size, healing time, and rate of healed patients healed after six months of follow-up.

## 2. Methods

### 2.1. Trial Design

An open-label randomized and controlled parallel clinical trial was performed involving 51 outpatients with complicated DFU that were admitted to specialized diabetic foot unit between November 2017 to December 2019. This study protocol received full approval from the local Ethics Committee of the Hospital Clínico San Carlos, Madrid, Spain (C.P.-C.I. 16/484-P). All patients provided written informed consent before inclusion. The present study was registered retrospectively in ClinicalTrial.gov (Registration no.: NCT04633642).

### 2.2. Participant

We enrolled patients in which the following inclusion criteria were implemented:

- Male and female patients over 18 years old
- Type 1 or type 2 diabetes with levels of HbA1c ≤ 85.8 mmol/mol (10%) within 30 days of the beginning of the study, based on a previous international, multicenter, randomized controlled trial [11]
- Wound stages IB, IIB, ID, and IID according to the University of Texas Diabetic Wound Classification [12]
- Wound duration of 1–24 months
- Wound size among 1–30 cm^2^ after debridement
- Diabetic foot ulcers showing mild or moderate infection, according to the criteria of the European Wound Management Association (EWMA) [13] and the Infectious Disease Society of America Guidelines [14]
- Ankle-brachial index (ABI) > 0.9 or ABI ≤ 0.9 and ankle systolic blood pressure (ASBP) ≥ 70 mmHg, or toe systolic blood pressure (TSBP) 50 mmHg. In patients with medial arterial calcification (ABI > 1.4) we considered Peripheral Arterial Disease (PAD) a toe–brachial index (TBI) < 0.7 [15,16]

We considered exclusion criteria:

- Chronic kidney disease (glomerular filtration rate < 60mL/min per 1.73 m^2^ during at least three months) or dialysis [17]
- Non-treated osteomyelitis
- Necrotizing soft tissue infections
- Critical limb ischemia patients with ABI ≤ 0.5 and ASBP < 70mmHg or < 50mmHg [15,16]
- Life expectancy < 6 months due to malignant DFU
- Pregnancy and lactation
- Patients diagnosed with human immunodeficiency virus (HIV) or hepatitis
- Patients showing local or systemic conditions that could impair tissue regeneration

### 2.3. DFU Assessment

A senior clinician in the management of diabetic foot (F.J.Á.-A.) always carried the baseline assessment of patients’ DFU. Sensorimotor neuropathy of DFUs was diagnosed using a biothesiometer (both from Novalab Iberica, Madrid, Spain) and Semmes-Weinstein 5.07/10g monofilament. Patients who did not feel one of the two tests were diagnosed with neuropathy [18,19].

Brachial and ankle systolic pressure were evaluated using a manual 8MHz Doppler (Doppler II, Huntleigh Healthcare Ltd., Cardiff, UK). Toe systolic pressure was taken via digital plethysmography (Systoe, Atys Medical, Madrid, Spain). Wound tissue oxygen levels were measured using transcutaneous oxygen readings (Radiometer Medical, Brønshøj, Denmark).

### 2.4. Intervention

#### 2.4.1. DFU Debridement and Wound Management

Patients were randomly assigned to receive either surgical debridement or UAW debridement every week during a six-week treatment period. All debridement procedures were performed by the same surgeon (J.L.L.-M.), who is specialist in the field of diabetic foot surgery with more than 21 years of experience.

Surgical debridement involved removal of all necrotic and devitalized tissue that was incompatible with healing, as well as surrounding callus.

UAW debridement was performed using an UAW SONOCA 185 device (Söring GmbH, Quickborn, Germany) by a senior clinician (J.L.L.-M.) with more than three years of experience applying this type of debridement. The UAW device is equipped with three UAW instruments with different sonotrode shapes and generates an ultrasound low frequency of 25 kHz. The choice of sonotrode depends on ulcer depth (ranges from superficial to deep). The UAW device piezoelectrically transforms the electrical energy delivered from the UAW device into mechanical oscillations in the sonotrode tip. In the majority of diabetic foot ulcers in the UAW group, a two-minute treatment with 40% intensity was made by holding the sonotrode in contact mode, holding it perpendicular to the wound bed and moving it across in an up-and-down pattern. For diabetic foot ulcers measuring > 15 cm^2^, the debridement treatment was increased to three minutes. In addition to UAW debridement, a scalpel was used for careful tissue removal, but only in cases where periwound tissue exhibited calluses or maceration. 

Between debridement sessions, sterile saline was used to clean all wounds prior to evaluation and all patients received standard of care for their diabetic foot ulcers, which consisted of moist wound dressings for wound management and proper off-loading (a removable walker cast based on the functioning and ambulatory status of the patient) as per the International Working Group of the Diabetic Foot guidelines [1,20]. When necessary, patients with moderate infections took empirical antibiotics during the treatment period, based on IDSA guideline recommendations [14], until the results from deep tissue culture were available [21]. After we received tissue culture results, we adjusted the antibiotic therapy to target the bacteria that were isolated during tissue culture. 

#### 2.4.2. Analysis of Tissue Samples

Soft tissue punch biopsies (3 mm) were taken after wound debridement sessions at week zero (day 0) and week six (day 42). After tissue collection, samples were immediately transported to the laboratory for cellular proliferation and microbiological analyses.

### 2.5. Outcome Measures

#### 2.5.1. Main Outcome Measure: Cellular Proliferation Analysis of Wound Tissue Samples

**Figure 1:**
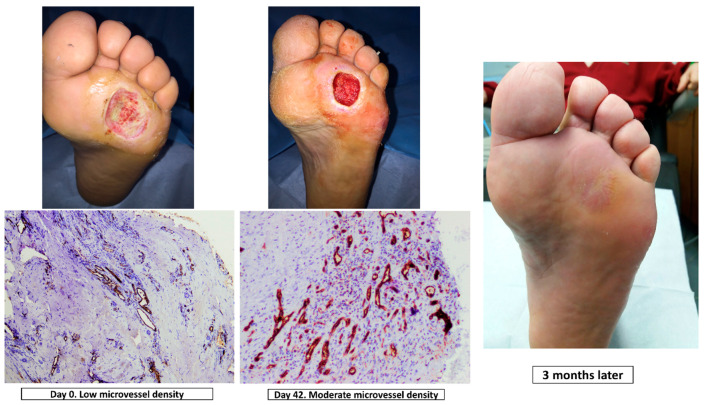
Immunohistochemistry staining of CD31 (magnification, ×10) at day 0 and day 42, clinical situation and progression of healing in a patient of the ultrasound-assisted wound (UAW) debridement group.

The same senior clinician interpreted all samples to evaluate the cellular proliferation. The microvascular structure of CD31, an endothelial marker, was subjected to immunohistochemical analysis and quantification to understand the effects of debridement on neo-angiogenesis, [22]. Sections of tissue sample were immunohistochemically stained with the CD31 marker. Light microscopy was used to count the number of microvessels/endothelial cells in a standardized grid, with the results expressed as microvessel density (Leica DMD 800 morphometric system). Microvessel density was scored in proportion to the following scale: 0 (absent), 1 (low, at least one microvessel), 2 (moderate) and 3 (more than two microvessels). (Figure 1).

**Figure 2:**
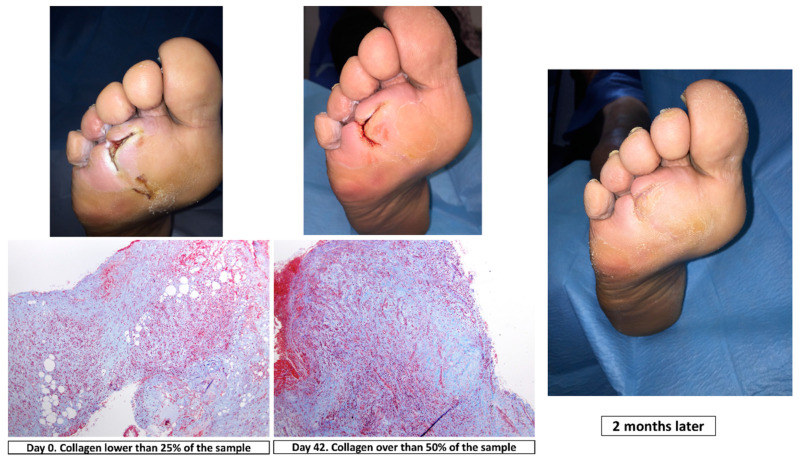
Masson’s trichrome staining (magnification, ×10) at day 0 and day 42, clinical situation and progression of healing in a patient of surgical group.

To differentiate collagen content from other components, such as muscle fibrin and erythrocytes, in tissue samples we used Massons’s trichome staining. Collagen content was scored according to the following scale: 0 (absent), 1 (mild), 2 (moderate) and 3 (severe) [23] (Figure 2).

**Figure 3:**
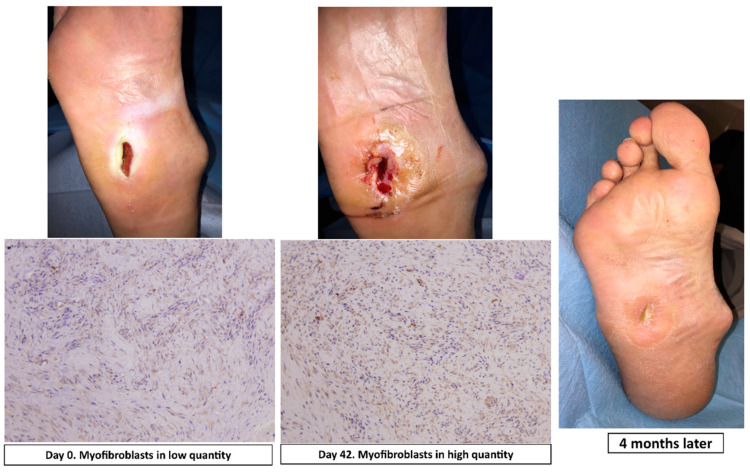
Actin staining (magnification, ×10) at day 0 and day 42, clinical situation and progression of healing in a patient of surgical group.

Actin staining was used to evaluate the presence of myofibroblasts involved in wound healing. These cells increase in number during wound healing. The number of stained cells was semi-quantitatively analyzed using a 0–3 scaling score (0 = no myofibroblasts, 1 = myofibroblasts in low quantities, 2 = myofibroblasts in moderate quantities, 3 = myofibroblasts in high quantities) (Figure 3).

#### 2.5.2. Secondary Outcome Measure: Microbiological Analysis of Wound Tissue Samples

Specimens of wound tissue were homogenized in 0.5 mL volumes of sterile phosphate buffered saline (PBS, Sigma Aldrich, St Louis, MO). After mechanical homogenization, the specimens were seeded in Columbia agar (BD, Sparks, MD), MacConkey agar (BD), Sabouraud dextrose agar (BD) and Columbia agar supplemented with nalidixic acid and colistin (BD) using a spiral plater workstation (Don Whitley Scientific, Shipley, UK). Quantitative and qualitative microbiological analyses were performed after incubation of plates at 37 °C for 24 h. Isolated microorganisms were identified by standard methods and susceptibility testing was performed in accordance with Clinical and Laboratory Standards by the disk diffusion method [24]. The results were expressed as CFU per gram of tissue (CFU/g) and the limit of detection was 10 colony-forming units (CFU).

#### 2.5.3. Third Outcome Measure: Evaluation of Wound Conditions

Diabetic foot ulcers were evaluated at patient admission and weekly before and after each debridement treatment. A validated wound scoring system was used to assess the wound bed tissue according to quality, presence and consistency of granulation tissue [25]. Furthermore, diabetic foot ulcers were evaluated for amounts of wound exudate and periwound skin conditions such as skin maceration by the same senior clinician (F.J.Á.-A.) according to the triangle wound assessment [26]. Wound healing was supervised weekly during the treatment period (6 weeks) and the wound size was assessed using Visitrak (Smith & Nephew, Hull, UK), determining the area of the lesion with an approximation of ± 5 mm^2^.

### 2.6. Follow-Up

Patients were followed up for 6 months after inclusion. During the follow-up period, we recorded ulcer healing. Ulcer healing was defined as complete epithelialization without any sustained drainage up to 24 weeks after the end of the study follow-up.

### 2.7. Sample Size

Granmo v.12 program (Municipal Institute of Medical Research, Barcelona, Spain) (https://www.imim.cat/ofertadeserveis/software-public/granmo/) was used to calculate the sample size. Thus, we analyzed 51 patients (24 in surgical group and 27 in UAW group) with a statistical power of 0.80 and an alpha of 0.05, with a power of the clinical difference of 37% to detect a statistically significant between groups.

### 2.8. Randomization

**Figure 4:**
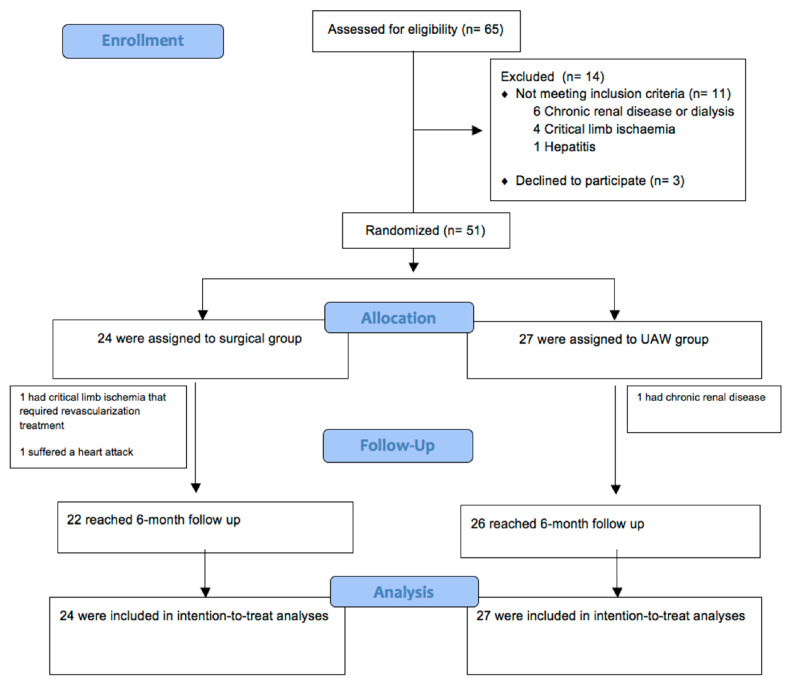
Study flow diagram.

A computer-generated random number table was used to carry out the randomization of the patients into the two groups by an investigator who was unaware of the identity of the participants. Figure 4 depicts the study flow diagram.

### 2.9. Blinding

None of the participants, care providers and outcome adjudicators were blinded to the interventions after assignment.

### 2.10. Statistical Analysis

Data were analyzed, based upon an intention-to-treat analysis, using the software package SPSS for IOs version 21.0 (SPSS, Inc. Chicago, IL, USA). Kolmogorov–Smirnov test was used to verify the assumption of normality of all continuous variables. Chi-square test was performed to calculate differences between groups and, if applicable, Fisher’s exact test for categorical variables. Student’s *t*-test and Mann–Whitney U test were performed for normally and abnormally distributed quantitative variables, respectively. Graphics to evaluate the differences among decrease in bacterial load and cellular proliferation between groups were done using GraphPad^®^ for Mac OS. 

We performed the study in accordance with the Declaration of Helsinki (2013 revision) and followed all regulations and local laws in clinical research investigations in patients [27]. 

## 3. Results

The surgical debridement group consisted of 24 patients and the UAW group consisted of 27 patients. Table 1 depicts clinical and demographic characteristics of both groups in our study population.

Compared to the surgical debridement group, patients in the UAW group were older (64.1 ± 12.4 years old vs. 58 ± 5.4 years old, *p* = 0.03), had longer diabetes durations (22 ± 12.9 vs. 10.3 ± 5.0, *p* = 0.001), and larger ulcers (7.47 ± 7.56 cm^2^ vs. 4.18 ± 3.32 cm^2^, *p* = 0.05). The UAW group also had a lower proportion of patients with moderate DFI (18.5% vs. 50%, *p* = 0.001) and antibiotic treatment (7.4% vs. 50%, *p* = 0.001).

Table 2 illustrates the features of the diabetic foot ulcers at patient admission (week 0, day 0) and at the end of the study (week six, day 42) in both groups.

After six weeks, Wollina scores improved significantly in both groups (surgical group: 2.5 ± 1.2—day 0 to 5.6 ± 0.7—day 42, *p* = 0.001 vs. UWA group: 2.15 ± 1.4—day 0 vs. 5.4 ± 1.5—day 42, *p* = 0.001), without any statistically significant differences among groups, *p* = 0.93). 

**Figure 5:**
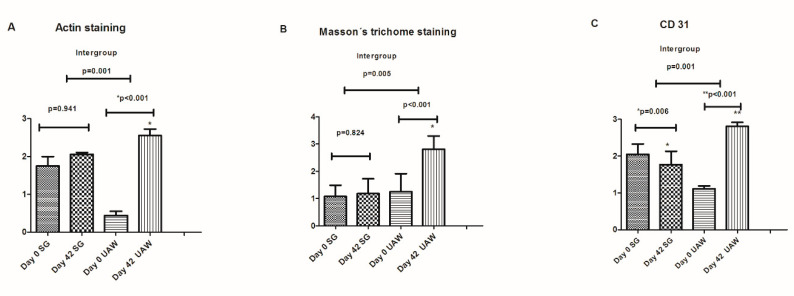
Cellular proliferation analysis. (**A**) Actin staining, (**B**) Masson’s trichrome staining and (**C**). CD31-positive vessels at day 0 and day 42 in both groups of our study population. SG: Surgical group. UAW: Ultrasound group.

Cellular proliferation improved significantly in the UAW group compared to the surgical group. (Figure 5).

**Figure 6:**
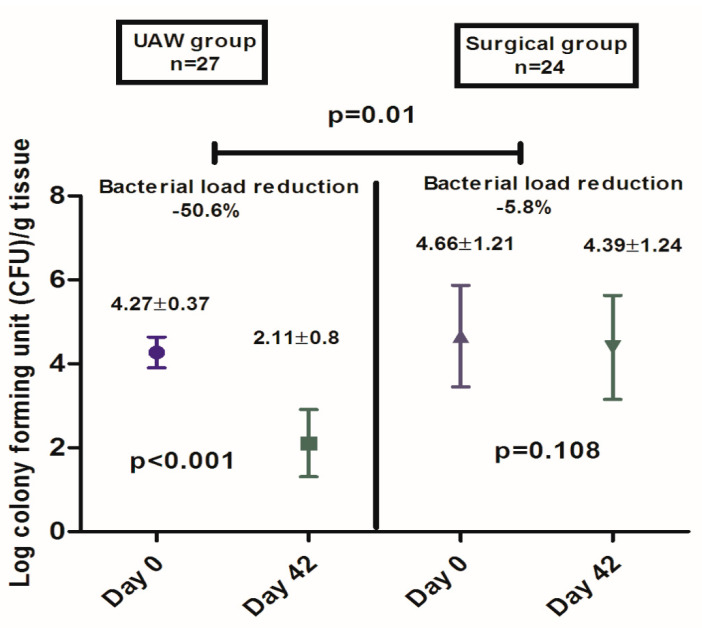
Comparison of bacterial loads in diabetic foot ulcer tissue samples at patient inclusion (Day 0) and after six-week treatment period (Day 42) after UAW or surgical debridement.

Bacteria load was also significantly reduced in the UAW group compared to the surgical group (UAW group 4.27 ± 0.37—day 0 to 2.11 ± 0.8 day— 42 vs. surgical group 4.66 ± 1.2—day 0 to 4.39 ± 1.24—day 42; *p* = 0.01) (Figure 6).

The rates of patients that were healed after 6 months of follow-up were similar in both groups (23 patients [85.1%] in the UAW group vs. 20 patients [83.3%] in the surgical group; *p* = 0.856). Time to healing was significantly lower (*p* = 0.04) in the UAW group (9.7 ± 3.8 weeks) than in the surgical group (14.8 ± 12.3 weeks).

## 4. Discussion

Based on CD31 staining, Masson’s trichrome staining and actin staining, this study demonstrates that patients with complicated diabetic foot ulcers treated with UAW debridement exhibit significantly improved cellular proliferation compared to patients receiving surgical debridement. We previously have proposed in a report from a closed panel meeting that increases in collagen, myofibroblasts and microvessel density following UAW debridement might result from the mechanical stimulation of neo-angiogenesis and fibroblasts at the wound site [4]. To our knowledge, this is the first randomized controlled trial (RCT) that has evaluated cellular proliferation and dermal repair in DFUs being treated with UAW debridement compared to DFUs receiving surgical wound debridement. The effects of UAW on cellular proliferation and dermal repair that we have observed in our study have also been described in preclinical studies involving diabetic mice. Maan et al. [28] found increased vascular endothelial growth factor, CD31 and stromal cell-derived factor 1 in the wound beds of noncontact, low frequency ultrasound-treated mice compared to controls. Likewise, Roper et al. [29] concluded that ultrasound therapy restores healing to diabetic animals by activating fibroblasts.

A previous single-center, non-comparative study showed that UAW debridement helped combat biofilm reformation and reduce bioburden in neuroischemic DFUs with mild infection [6]. In this study, patients did not receive systemic antibiotics and the authors found that mean bacterial load in wound tissue samples before and after wound debridement after treatment period was log 5.55 ± 0.91 CFU/g and log 4.59 ± 0.89 CFU/g, respectively (*p* < 0.001). In the current study, we observed that bacteria load was significantly reduced in the UAW group compared to the surgical group (UAW group log 4.27 ± 0.3—day 0 to log 2.11 ± 0.8 CFU/g—day 42 vs. surgical group log 4.66 ± 1.21 CFU/g day 0 to 4.39 ± 1.24 CFU/g day 42; *p* = 0.01). The main differences between the previous study and our current study were that our study population included patients with moderate infection and when necessary, these patients took empirical antibiotics that were selected according to IDSA guidelines [12]. Further, antibiotics were switched when needed to target bacteria that were detected from deep tissue cultures [17]. As such, systemic antibiotics likely promoted the reduction of bacterial loads observed in our study. Notwithstanding, the UAW groups had a lower proportion of patients that took antibiotics than the surgical group (*n* = 2, 7.4% vs. *n* = 12, 50%, *p* = 0.001). Furthermore, we observed that after the treatment period (six weeks), Wollina scores improved in both groups, and the UAW group exhibited significant improvements in terms of periwound skin and exudate levels (see Table 2). In this regard, our results are in agreement with the statement that UAW debridement is as effective as surgical debridement in removing bacteria while selectively removing affected tissue and protecting intact tissue at the wound site [6,30]. Furthermore, similar rates of patients were healed after six months of follow-up in both groups. These results are consistent with a recent systematic review to compare the effect of UAW versus nonsurgical sharp debridement, where no difference in healing outcomes between both debridement treatments of diabetic foot ulcers was found [31]. In our study population, the time it took for ulcers to heal was significantly shorter in the UAW group than in the surgical group. In a RCT by Michailidis et al. [32], they observed faster healing in DFU patients receiving non-surgical sharps debridement versus patients receiving UAW debridement. However, their results are unable to be generalized due to the small sample size.

As previously mentioned, this is the first RCT to evaluate the effects of UAW debridement and surgical wound debridement on cellular proliferation and dermal repair in ulcers and demonstrate that UAW debridement significantly improves cellular proliferation compared to surgical debridement. Importantly, our study also demonstrated that UAW debridement is as effective as surgical debridement in removing affected tissue, protecting intact tissue at the wound site. As such, UAW debridement could be an effective alternative to surgical debridement when it is contraindicated or not available for use on patients with DFU. Our experience also indicates that UAW therapy is appropriate for serial debridement of DFU patients with poor vascular statuses (neuroischemic aetiologies), anticoagulants prescriptions, and deteriorating wound beds likely infected with biofilms [4,6].

The main limitation of our study was the difference in baseline characteristics among groups of our study population, such us duration of diabetes diagnosis, glycated hemoglobin, Texas Classification or type of infection (mild/moderate infection) (see Table 1). Further studies should include a more homogenous population in both groups to confirm our findings. We consider that future trials may also evaluate efficiency or cost-effectiveness of both treatments and the possibility of the spreading of solution and microbes (aerosols) that may occur during UAW debridement and how that may be affected depending on the flow intensity of the solution and the device used to contain the fluid.

## 5. Conclusions

Patients with complicated diabetic foot ulcers that received UAW debridement showed a significant improvement in cellular proliferation and reduction in bacterial load after six weeks of treatment compared to patients receiving surgical debridement. Wollina wound scores improved in both groups after six weeks. The numbers of patients that were healed after six months were similar between the two groups. However, the time it took for ulcers to heal was shorter in the UAW group compared to the surgical group. Patients who had undergone UAW debridement had better bioburden control and cellular proliferation, which may explain the lower time needed to heal in this group.

We conclude that UAW debridement could be an effective alternative when surgical debridement is not available or is contraindicated for use on patients with DFU.

## Figures and Tables

**Table 1:** Clinical and demographic characteristics of the study population.

	Patients Included *n* = 51		
	Surgical Group (*n* = 24)	UAW Group (*n* = 27)	*p*-Value
**Age (years)**	58 ± 5.4	64.1 ± 12.4	0.03
**Male/Female, *n* (%)**	24(100)/0	24 (88.8)/3 (11.2)	0.09
**Type 1/Type 2 DM *n* (%)**	0/24 (100%)	5 (18.5%)/22 (81.5%)	0.02
**Duration of diabetes diagnosis, mean ± SD**	10.3 ± 5.0	22 ± 12.9	0.001
**Glycaemia (mmol/L), mean ± SD**	7.68 ± 2.62	8.79 ± 3.19	0.18
**Glycated hemoglobin mmol/mol, mean ± SD**	51 ± 4.5	57 ± 9.9	0.09
**Mean wound evolution (weeks) mean ± SD**	7.33 ± 8.95	8.63 ± 7.81	0.58
**Mean Ulcer area cm^2^, mean ± SD**	4.18 ± 3.32	7.47 ± 7.56	0.05
**Texas Classification** **IB** **IIB** **ID** **IID**	4 (16,7) 8 (33,3) 8 (33,3) 4 (16,7)	4 (14.8) 3 (11.1) 12 (44.4) 8 (29.6)	0.001
**Mild/Moderate infection, *n* (%)**	12 (50%)/12 (50%)	22 (81.5%)/5 (18.5%)	0.001
**Antibiotic treatment, *n* (%)**	12 (50%)	2 (7.4%)	0.001

Data are shown as *n* (%), as mean ± SD: standard deviation or mean (Q1:1st quartile; Q3: 3rd quartile).

**Table 2:** Diabetic foot ulcers characteristics at week 0 (inclusion in the study, day 0) and at week six (day 42) in both groups of our study population.

Variable	Surgical Group (*n* = 24)		*p*-Value	UAW Group (*n* = 27)		*p*-Value	*p*-Value Inter-Group
	Day 0	Day 42		Day 0	Day 42		
**Ulcer area (cm^2)^**, **Mean (SD)**	4.18 ± 3.32	0.88 ± 1.04	<0.001	7.47 ± 7.56	1.00 ± 1.22	<0.001	0.711
**Periwound skin *n* (%)**							
**Healthy**	4 (16.7%)	8 (33.3%)		8 (29.6%)	20 (74.1%)		
**Macerated**	16 (66.7%)	16 (66.7%)	0.05	15 (55.5%)	3 (11.1%)	<0.001	0.001
**Hyper-keratosis**	4 (16.7%)	0		3 (11.1%)	3 (11.1%)		
**Hyperemic**	0	0		1 (3.7%)	1 (3.7%)		
**Exudate levels *n* (%)**							
**Absent**	0	4 (16.7%)		1 (3.7%)	7 (25.9%)		
**Low**	8 (33.3%)	8 (33.3%)	0.22	5 (18.5%)	16 (59.3%)	0.009	0.05
**Medium**	16 (66.7%)	12 (50%)		20 (74.1%)	3 (11.1%)		
**High**	0	0		1 (3.7%)	0		
**Tissue types on the wound bed**							
**Granulated**	10 (41.6)	18 (75%)		4 (14.8%)	23 (85.2%)		
**Hyper-granulated**	4 (16.7%)	0	<0.001	4 (14.8%)	0	<0.001	0.61
**Slough**	10 (41.6%)	6 (25%)		19 (70.4%)	4 (14.8%)		
**Necrotic**	0	0		0	0		
**Wollina score ± SD**	2.5 ± 1.2	5.6 ± 0.7	<0.001	2.15 ± 1.4	5.4 ± 1.5	<0.001	0.93

Data are shown as *n* (%), or as mean ± SD (standard deviation).
